# Impact of Intensive Glycemic Treatment on Diabetes Complications—A Systematic Review

**DOI:** 10.3390/pharmaceutics15071791

**Published:** 2023-06-22

**Authors:** Juliana Poonoosamy, Philippe Lopes, Priscille Huret, Randa Dardari, Alfred Penfornis, Claire Thomas, Dured Dardari

**Affiliations:** 1 LBEPS, IRBA, Université Paris Saclay, 91025 Evry, France; 2 Al Fourkan Diabetes Center, Al Fourkan, Aleppo, Syria; 3 Diabetology Department, Centre Hopitalier Sud Francilien, 91100 Corbeil-Essonnes, France; 4 Paris-Sud Medical School, Paris-Saclay University, 91100 Corbeil-Essonnes, France

**Keywords:** HbA1c, cardiovascular events, type 2 diabetes, micro- and macrovascular diabetes complications

## Abstract

Diabetes complications can be related to the long duration of the disease or chronic hyperglycemia. The follow-up of diabetic patients is based on the control of chronic hyperglycemia, although this correction, if obtained rapidly in people living with severe chronic hyperglycemia, can paradoxically interfere with the disease or even induce complications. We reviewed the literature describing the impact of the rapid and intense treatment of hyperglycemia on diabetic complications. The literature review showed that worsening complications occurred significantly in diabetic microangiopathy with the onset of specific neuropathy induced by the correction of diabetes. The results for macroangiopathy were somewhat mixed with the intensive and rapid correction of chronic hyperglycemia having a neutral impact on stroke and myocardial infarction but a significant increase in cardiovascular mortality. The management of diabetes has now entered a new era with new therapeutic molecules, such as gliflozin for patients living with type 2 diabetes, or hybrid insulin delivery systems for patients with insulin-treated diabetes. Our manuscript provides evidence in support of these personalized and progressive algorithms for the control of chronic hyperglycemia.

## 1. Introduction

Diabetes is a worldwide chronic disease characterized by high blood glucose levels [1]. About 422 million people around the world suffer from diabetes, and its prevalence has continued to increase gradually over the past few years [2]. Diabetes can lead to irreversible complications, including kidney failure, heart failure, leg amputation, nerve damage, and vision loss. Many people with diabetes may experience reduced blood flow and nerve damage in the feet, leading to foot ulcers or even amputation [3]. Diabetes may also cause both macroangiopathy and microangiopathy. The macrovascular and microvascular complications of diabetes have similar pathogenetic backgrounds despite affecting both small and large vessels [4]. 

Rapidly improving glycemic control seems to decrease the risk of microvascular complications in people living with diabetes, and several randomized clinical trials have shown the benefits of the intensive correction of hyperglycemia on the macrovascular consequences of diabetes. This literature review aims to provide a better understanding of the effect of the intensive and rapid correction of hyperglycemia on different macrovascular outcomes such as myocardial infarction (MI), stroke, arteriopathy of the lower limbs, and cardiovascular mortality, as well as microvascular complications such as treatment-induced neuropathy, retinopathy, renal dysfunction, and Charcot’s neuroarthropathy.

## 2. Methods

**Figure 1:**
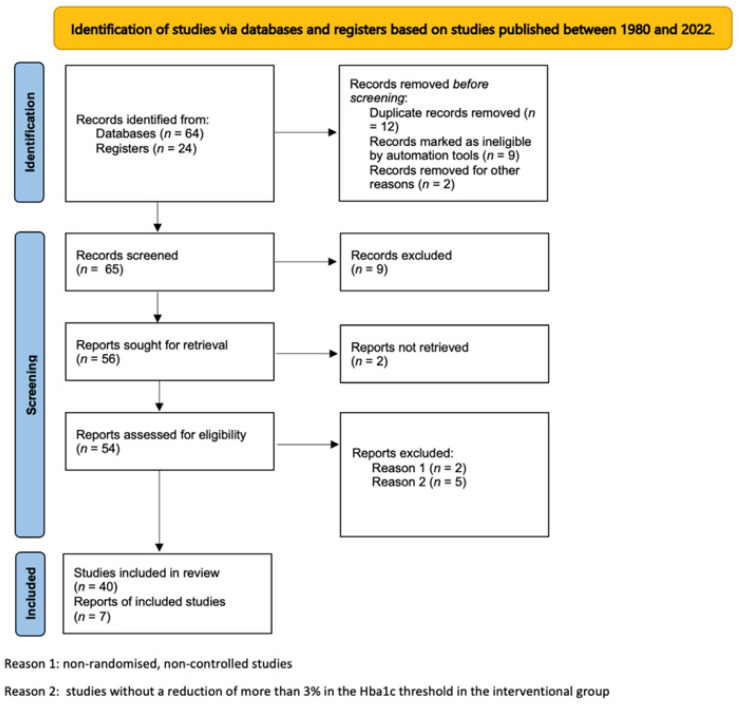
Flowchart with information on the results of the research carried out in our review.

A literature review was conducted based on manuscript searches with the following indicators: diabetes complications and intensive glycemic treatment. We conducted our review by searching the flow terms: diabetic people, intensive glycemic therapy, control group, and micro and macrovascular diabetes’s complications. Intensive glycemic therapy was defined as a decrease of at least three points in the Hba1c levels over the duration of the subject’s participation in the study. It has been stated that the reduction in the HbA1c levels is achieved by the introduction of an antidiabetic treatment. This allowed us to define equally the control group whose participants did not benefit from this reinforcement. The manuscript search used several electronic databases, such as MEDLINE, EMBASE, Cochrane, the Central Register of Controlled Trials, and PubMed based on studies published between 1980 and 2022. The results are presented below according to the type of complications. Only publications linked to the objective of this study were selected, namely the impact of intensive glycemic treatment on diabetes complications. We established a common criterion for selecting the studies, namely the clear definition of two randomized groups of patients: one group whose HbA1c remained stable and another whose HbA1c significantly decreased. The extracted data included the characteristics of the participants (sex, age, duration of diabetes, etc.), their HbA1c evolution, and its impact on diabetes-related complications. The results obtained from non-randomized controlled trials (RCTs) were excluded, except for the results recovered for treatment-induced neuropathy in diabetes and for renal dysfunction due to the small number of trials that assessed the two complications. Randomized trials showing a reduction in the Hba1c levels of less than 3% were equally excluded. The studies included ithe quantitative synthesis (meta-analysis) based on the RCTs reports were also included. We used the Prisma statement instructions in the bibliographic search plan [5]. Figure 1 explains the flowchart that we used to achieve the results cited in our review.

## 3. Results

### 3.1. Macroangiopathy 

Several randomized clinical trials (RCT) investigated the effects of intensive hyperglycemia treatment compared to conventional treatment on the abovementioned macrovascular complications in patients living with diabetes, namely, stroke, myocardial infarction, cardiovascular mortality, and arteriopathy of the lower limbs. Among these RCTs, we focused on the following five: the UK Prospective Diabetes Study (UKPDS), the Prospective Pioglitazone Clinical Trial in Macrovascular Events (PROactive), the Action to Control Cardiovascular Risk in Diabetes (ACCORD), the Action in Diabetes and Vascular Disease Controlled Evaluation (ADVANCE), and the Veterans Affairs Diabetes Trial (VADT). We also chose to include other trials with random meta-analyses in order to gain a better understanding of the effect of intensive glucose-lowering therapy compared to conventional therapy. 

#### 3.1.1. Stroke

People with diabetes mellitus have a higher risk for incident stroke [6]. Recent evidence from large-scale RCTs [7] showed that the intensive control of hyperglycemia is not significantly more effective than conventional treatment in reducing the rate of stroke. A large meta-analysis of nine RCTs [8], with 6224 patients in the intensive treatment group and 6273 patients in the non-intensive treatment group, found 331 cases of stroke in the intensive group (5.3%) versus 319 in the non-intensive group (5.1%) (*p* = 0.69). This study showed no significant differences in the reduction in stroke incidence, with just a small decrease in the group undergoing intensive hyperglycemia management [8]. The authors reported that this might have been due to the reduction in or balancing of residual factors such as the lipid, blood, or homocysteine levels. However, the same meta-analysis showed that the intensive correction of hyperglycemia could reduce the risk of stroke in obese patients with a body mass index exceeding 30 kg/m^2^. For the authors, this might have been due to the higher glucose concentrations, which always promoted a high blood viscosity and, therefore, increased the risk of vascular complications. Conversely, the previous RCTs reported in a meta-analysis [9] showed that stroke incidence was not significant in the intensive hyperglycemia treatment group. Another meta-analysis of epidemiologic studies [10] reported that lower glucose levels could reduce the risk of ischemic stroke, hemorrhagic stroke, and unclassified stroke. Furthermore, in the ACCORD RCT [11], 10,251 patients with a median diabetes duration of 10 years were randomized to receive intensive glucose-lowering treatment. The intensive treatment group was aimed at hemoglobin A1C (HbA1c) < 6.0%, whereas the standard group targeted the HbA1c levels in the range of 7.0–7.9%. After treatment, the HbA1c levels decreased to 6.4% and 7.5% in the intensive and standard groups, respectively. This study showed no significant effect from the rapid correction of hyperglycemia on the risk of stroke. In the UKPDS study [12], the patients receiving metformin, insulin, and sulfonylureas compared to the patients undergoing conventional treatment had a lower risk for all the diabetes endpoints (−30%, *p* = 0.020), including stroke. In the PROactive RCT [12], which included 5238 patients with macrovascular disease, there was a significant decrease in both fatal and non-fatal stroke among the patients treated with pioglitazone with a history of stroke (*n* = 76/2605 in pioglitazone vs. *n* = 96/2633 in placebo, *p* < 0.05) [13].

#### 3.1.2. Myocardial Infarction

The UKPDS RCT [11] included 4203 patients with newly diagnosed diabetes and the follow-up was 10 years. The intensive hyperglycemia treatment group had a median HbA1c level of 7.0% during the follow-up compared to 8.2% in the conventional group. The authors observed a significantly lower incidence of myocardial infarction of 12% in in the intensive treatment group (*p* < 0.05). The reduction in fatal and non-fatal MI was almost significant, with 6.8 events per 1000 patient-years in the intensive treatment group versus 9.9 events per 1000 patient-years in the conventional group (*p* = 0.052). In the same study, the patients treated with metformin had a median HbA1c of 7.4% during the follow-up compared to the patients undergoing conventional treatment, who had a median HbA1c of 8.0%. The metformin-treated patients also had a 32% reduced risk for all the diabetes endpoints, including a 39% reduction in fatal and non-fatal MI. In the PROactive RCT [13], there were no significant differences in the primary endpoints, including all-cause mortality, stroke, non-fatal MI, and acute coronary syndrome.

Nevertheless, a significant reduction in the secondary endpoints, including infarction, was observed in the patients who received pioglitazone (90/2605 versus 116/2633 in the control arm; *p* < 0.005) [13]. The primary objective of the ACCORD trial [10] was to investigate whether major CV events in individuals with type 2 diabetes could be prevented using intensified glucose control (target HbA1c 6.0% vs. 7.0–7.9%). The participants in the ACCORD trial were enrolled between 2001 and 2005, and the main outcomes were reported in 2008 with a median follow-up of 3.5 years to December 2007. The principal enrolment requirements were that the patients had type 2 diabetes with an HbA1c ≥ 7.5% and an elevated CV risk. The elevated cardiovascular risk was identified as (1) patients aged between 40 and 79 years with a prior record of cardiovascular illness; (2) patients aged 55–79 years with a history of atherosclerosis, albuminuria, or left ventricular hypertrophy; or (3) patients aged 55–79 years with at least two cardiovascular risk factors (dyslipidemia, high blood pressure, present smoking, or obesity). The major exclusions criteria included frequent or recently occurring episodes of severe hypoglycemia, failure to use home glucose testing or insulin injection, a body mass index >45 kg/m^2^, blood creatinine >132.6 µmol/L (1.5 mg/dL), or other serious illnesses. To avoid potential confusion between the glycemic control and CV events, the participants with a major cardiovascular event within the first two years of therapy were removed from the analyses. A total of 9752 patients were enrolled in the definitive analysis. In the ACCORD study, the authors analyzed groups of HbA1c trajectories independently in the standard and intensified treatment groups using a latent class growth paradigm. The adjusted HR for CV death, non-fatal myocardial infarction, non-fatal stroke, and heart failure used group 1 (HbA1c change from 7.8 ± 0.8% at baseline to 7.0 ± 0.6% at 2 years) as the reference group. The patients in group 5 had a reduced risk of non-fatal stroke (HR 0.423, 95% CI 0.190–0.942, *p* = 0.035) when compared to group 1. However, there was no statistically significant difference between the groups in the risk of cardiovascular death and other outcomes. In contrast, the individuals in groups 3, 4, and 8 had poorer results than those in group 1. Similar results were seen even after removing participants who developed the primary composite outcome within the first two years of therapy.

In the ADVANCE RCT [14], 11,140 patients with a diabetes duration of 8 years received either intensive glucose-lowering treatment (HbA1c < 6.5%) or conventional treatment with HbA1c at 7.3%. The patients were followed for up to 5 years. The authors observed a significant reduction of 10% for the primary endpoint, including both microvascular and macrovascular events such as non-fatal MI. The VADT RCT [15] included 1791 patients who received intensive glucose-lowering treatment or standard treatment. The intensive group aimed for HbA1c < 6%, whereas the standard treatment targeted HbA1c < 9%. The intensive and standard groups achieved HbA1c levels of 6.9% and 8.4%, respectively. After a median follow-up of 5.6 years, the reduction in the primary endpoints, including MI, was not significant in the intensive treatment group [15].

On a smaller scale in a retrospective study, another recent report also showed a clear increase in myocardial infarction in subjects with a recent dramatic reduction in HbA1c vs. non-dramatic reducers [16].

#### 3.1.3. Cardiovascular Mortality

In the UKPDS trial [12], there was no significant reduction in all-cause mortality, including cardiovascular death, in the intensive treatment group. Cardiovascular disease accounted for 62% of the total mortality in overweight patients in the conventional treatment group. The metformin (intensive group) group had a 36% lower risk (*p* = 0.011) of all-cause mortality than the conventional group [12]. In the same study, the patients randomized to the intensive treatment group who received metformin, insulin, and sulfonylureas had a lower risk of reaching the endpoints, including cardiovascular death. However, in the ACCORD trial [6], the study was stopped after 3.5 years of follow-up in 2008 due to a 35% increase in cardiovascular mortality in the patients undergoing intensive glycemic control [6]. The subgroup analysis showed a more significant positive effect on the primary endpoint in the patients without cardiovascular disease and with better diabetes control (HbA1c < 8%) who received the intensive treatment. Furthermore, hypoglycemia requiring assistance and weight gain exceeding 10 kg were significantly higher in the intensive therapy group, which indicated that intensive hyperglycemia management could be fatal, as this treatment did not significantly reduce the major cardiovascular events but rather increased mortality. In the ADVANCE trial [14], after a median follow-up of 5 years, no significant increase was observed in cardiovascular mortality. As mentioned above, the UKPDS, PROactive, and ADVANCE trials showed no significant trends in decreasing cardiovascular mortality in the intensive treatment group. Nevertheless, in the ACCORD trial, intensive glycemic control increased cardiovascular death, and it was associated with increased mortality in the VADT trial [17]. In another meta-analysis, including 12 trials on cardiovascular mortality, the authors found no significant decrease in cardiovascular mortality in the group receiving the intensive intervention [17].

In the REACT trial, a multicentric trial conducted between 2010 and 2013 in Brazil, 5006 individuals were enrolled and evaluated. Of the 5006 patients, significant clinical outcomes were found in 332 subjects with a decrease in the Hba1c levels during a one-year monitoring period [18].

The association between increased cardiovascular mortality and Hab1c reduction or intensive treatment was equally described in a recent meta-analysis, which included the results of 14 clinical trials on cardiovascular outcomes. The mean population size was 9401, the mean age was 64 years, the mean age at diabetes diagnosis was 12 years, and the median follow-up time of the study was 120 weeks [19].

#### 3.1.4. Arteriopathy of Lower Limbs

Vascular surgery guidelines suggest that intensive glycemic control (HbA1c < 7%) should be targeted to reduce the risk of lower limb amputation [20]. These guidelines require HbA1c levels of <7% or even >6% in patients with diabetes associated with peripheral arterial disease [20]. Furthermore, in the ACCORD clinical trial [21], the authors examined the relationship between glycemic control and the incidence or recurrence of lower-extremity amputation in patients with type 2 diabetes, finding that intensive glycemic control was associated with a reduced risk of lower-extremity amputation. The study suggested that intensive glycemic control could reduce the risk of limb amputation, even over a short period of time. 

### 3.2. Microangiopathy 

Microvascular complications frequently occur in people with diabetes, leading to a substantial increase in morbidity and a considerable decrease in the quality of life [22].

### 3.3. Treatment-Induced Neuropathy in Diabetes

Hyperglycemia causes a series of chemical changes in the body that eventually lead to nerve damage. This damage, which can range from functional damage (slowing of electrical conduction) to structural damage of the nerves, is increasingly difficult to reverse. Diabetes can affect all the nerves in the body [23,24]. It primarily affects two types of nerves: the peripheral nerves that control muscles and feeling in the skin and the nerves of the autonomic nervous system that control the functioning of the viscera. Neuropathy, also known as cardiac autonomic neuropathy (CAN), is a long-term complication of type 2 diabetes. Its prevalence ranges from 31% to 73% in people with type 2 diabetes [17], with no difference in prevalence between men and women [25]. High glucose levels in the blood can damage the small blood vessels supplying the nerves, which are then deprived of important nutrients. This can in turn damage the nerve fibers (axons) [26,27]. 

The concept of treatment-induced diabetic neuropathy (TIDN) is quite new. In fact, the first description of the disorder was established in 1933, when a diagnosed in a lady living with diabetes showed intense discomfort after starting insulin treatment [28]. Insulin was stopped and the was pain quickly resolved. A further attempt at insulin in the same case led to a rapid onset of a burning sensation. At that time, an insulin allergic reaction was suggested and the term “insulin neuritis” was used. The description of insulin neuritis has been used in a few case reports describing similar clinical findings over the last 80 years. The most usual clinical presentation is the abrupt appearance of intense pain in the context of an improved glycemic regulation [29,30]. The literature, however, also contains other similar cases referred to as acute painful neuropathy, diabetic neuropathic cachexia, or other similar types [31,32].

In general, the overall characteristics of these cases are similar. People with type 1 diabetes and a history of poor glycemic control present a rapid improvement in glycemic control, usually due to insulin treatment. Then, a few days or a few weeks after glycemic control, severe burning and stabbing pains appear locally or diffusely [33,34]. Gibbons [35] described for the first time 16 people with diabetes who developed neuropathic pain after an improvement in blood glucose control. Thus, the term “treatment-induced diabetic neuropathy” (TIDN) was suggested as a more accurate equivalent to “insulin neuritis”. It was also noted that people with TIDN developed autonomic neuropathy in addition to painful peripheral neuropathy. Nephropathy and proliferative retinopathy also concomitantly developed, suggesting a diffused microvascular process [36].

A number of hypotheses have been put forth to understand the pathophysiology of TIDN. A potential explanation is the onset of “relative” hypoglycemia in an individual with chronic, persistent high glucose levels, which results in a breakdown of energy-dependent axonal trafficking [32]. A well-controlled trial of modest hypoglycemia in humans using an insulin clamp led to the appearance of tactile hyperalgesia and transient autonomic failure related to the discharge of pro-inflammatory cytokines [37]. The other mechanisms that were suggested include the generation of an arteriovenous shunt, leading to endoneural ischemia and regenerative nerve fiber discharge [32,36]. The study of an animal model of TIDN may shed light on the potential pathophysiological mechanisms of the disease [32]. The prevalence of TIDN in the general population is unknown. 

Several abstracts presented at recent international meetings highlighted the scientific interest in this phenomenon, although the results of these studies have not yet been published. Thus, the only available data are based on smaller non-population-based studies or secondary results gleaned from larger clinical trials that include other microvascular outcomes of overlapping interest. Gibbons and Freeman [38] conducted a review of all the records of patients with TIDN over a period of 5 years. To standardize the operational definition of TIDN, the subjects included in the study had to meet the following criteria: (1) the onset of neuropathic pain or autonomic dysfunction within 8 weeks of a decrease in the mean blood glucose; (2) neuropathic pain of at least three points on a 10-point Likert scale or severe autonomic dysfunction requiring medical attention; and (3) a change in the glycemic control resulting in a decrease in HbA1C of at least two percentage points over a 3-month period. 

Over a 5-year period, 954 patients were evaluated for a diagnosis of TIDN, of whom 104 (10.9% of the total number evaluated) met the criteria for TIDN. The number of patients with probable TIDN in this study, which was conducted at two registered sites in a single medical center, was greater than the total number of patients reported in the previous 80 years. This observed frequency of TIDN challenged the presumption that it is a rare disorder [39]. 

### 3.4. Microvascular Complications Associated with TIDN

As noted in the original description of TIDN [40], a number of other microvascular manifestations can occur in addition to autonomic and peripheral neuropathy [27]. As explained above, the development of retinopathy is a common comorbid complication occurring in people who have developed TIDN. Before the reduction in their HbA1C levels, less than half of the group with TIDN suffered from retinopathy, while only 3% of individuals had proliferative retinopathy. After developing TIDN, however, 90% of the group had severe non-proliferative or proliferative retinopathy [41].

#### 3.4.1. Retinopathy

In the diabetic ophthalmology based research literature, the Diabetes Control and Complications Trial (DCCT) found that a small group of people developed diabetic retinopathy (DR) after the initiation of aggressive glucose control [37,38,39]. This was described as “early worsening of retinopathy”. Since these studies in the 1980s, a premature progression of retinopathy has been documented in people with type 1 and type 2 diabetes [42]. In the initial DCCT study, an early progression of retinopathy occurred in 22% of the intensive treatment group compared to only 13% in the standard treatment group. Rapid glycemic improvement may, indeed, worsen DR [38] In special contexts, such as the initiation of continuous subcutaneous insulin delivery [43] or during pregnancy [44], the deterioration of DR was recorded in a small number of cases. More consistently, for type 1 diabetes, the DCCT has shown an early progression of DR during the first year of intensive glucose management [45]. For type 2 diabetes, there have been a few reports of early DR worsening after bariatric surgery [46] or in minorities [47]. Recently, there have been some concerns regarding the aggravation of DR following the high rate observed in semaglutide-treated subjects in the SUSTAIN-6 trial [48], including five cases of blindness versus one in the placebo arm, which seemed to be related to the simagnitude and speed of the HbA1c lowering rather than the semaglutide itself [49]. It might be helpful to forecast the risk of DR using clinical data, including past HbA1c trends. The extent of the HbA1c lowering has been positively related to the risk of DR advancement in many studies, as recently analyzed [43,50]. 

#### 3.4.2. Renal Dysfunction 

Only a few studies evaluated the impact of intensive antidiabetic therapy on the impairment of renal function and diabetic nephropathy. Renal function is also impaired in people with TIDN. Microalbuminuria was detected in 17% of cases before the development of TIDN. By contrast, 1 year after the onset of TIDN, microalbuminuria was detected in 84% of individuals during testing. It should be noted that microalbuminuria is a poor proxy for kidney function, as it is rather an indication of kidney damage. A small group of people (8%) with TIDN had a significant increase in serum creatinine and required hemodialysis in some cases [41,42]. More recently, in a small cohort, a clear degradation of renal function was identified during the rapid correction of chronic hyperglycemia [51]. 

#### 3.4.3. Charcot’s Neuroarthropathy

Charcot’s neuroarthropathy (CN) is a devasting complication of the joints occurring in people living with diabetes. In CN, bone modeling factors such as RANKL (receptor activator of nuclear factor-κB ligand) and its natural antagonist osteoprotegerin (OPG) play an important role in the development of this rare diabetes-related complication [52]. The pathophysiology of CN is still not fully understood. However, the diagnosis of CN has recently been described in situations that resemble the intensive correction of hyperglycemia, such as a simultaneous kidney–pancreas transplantation [53,54,55]. In two cohorts [56,57], it was retrospectively demonstrated that a significant reduction in the HbA1c levels occurred 3 and 6 months prior to the discovery of CN, while the high rate of CN after simultaneous pancreas–kidney transplantations reinforced the idea that the rapid reduction in the HbA1c levels plays a key role in the onset of CN [57,58]. The patients who developed CN following a double pancreas–kidney transplantation experienced a rapid and significant reduction in the Hba1c levels, in which pancreatic function was restored following the transplantation. The potential role played by intensive glycemic reduction in the development of CN can be explained as follows. Osteoprotegerin is inhibited by the reduction of Hba1c [59], which induces an increase in the RANKL levels due to the decrease in its antagonist. Although CN coincides with joint trauma, the bone shaping factors are nevertheless disrupted, including the rapid maturation of the osteoblast, leading to bone lysis.

**Figure 2:**
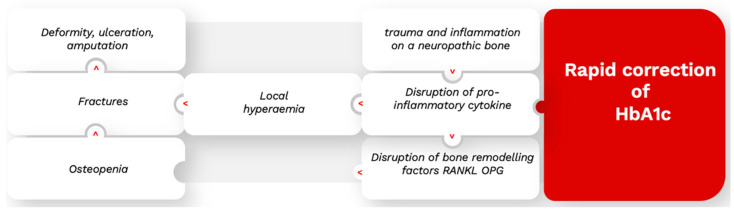
Impact of the rapid correction of HbA1c in CN physiopathology.

It, therefore, follows that the precursor to the onset of CN is the rapid reduction in Hba1c in the subjects with uncontrolled diabetes, although this must be concomitant with repetitive trauma to the joints (Figure 2).

## 4. Discussion

Based on our literature review, the intensive and rapid correction of hyperglycemia may impact the risk of macrovascular outcomes and all-cause mortality. We reviewed a meta-analysis [60] combined with five large RCTs (i.e., UKPDS, ADVANCE, ACCORD, VADT, and PROactive) that included a total of 32,649 patients, of which 17,267 received intensive glycemic control therapy. The authors investigated the effect of intensive glucose therapy on cardiovascular mortality, non-fatal MI, stroke, and arteriopathy of the lower limbs. The reduction in non-fatal MI in the groups receiving the intensive treatment was highly significant. As for non-fatal stroke, its incidence did not decrease in the intensive treatment group. There was no significant reduction in cardiovascular mortality when the results of the five trials were combined, although the ACCORD trial evidenced a highly significant increase in cardiovascular deaths [60]. This finding may have been due to the aggressive treatment involving four to five glucose-lowering drugs, which induced more hypoglycemic events as well as significant weight gain in these patients. Indeed, compared to the cholesterol or blood pressure-lowering studies, the positive effect of glucose control might be observed after a longer duration, whereas it was normally observed in a 5-year treatment period in the RCTs. In fact, the Diabetes Control and Complications Trial/Epidemiology of Diabetes Interventions and Complications (DCCT/EDIC) [60,61] and UKPDS studies showed that the effect on the prevention of macrovascular events became significant after 10 years of follow-up despite the lack of glycemic control during the follow-up period. Furthermore, during the follow-up period of the UKPDS and Steno 2 trials, better glycemic control had a positive effect on all-cause mortality [59,60]. The authors also suggested that this delayed positive effect could be due to the progressive and long-term glycemic control during the trials, which prevented the development of neuropathy associated with a higher incidence of cardiovascular disease. 

However, the Action to Control Cardiovascular Risk in Diabetes Study Group [62] included 10,251 patients with a median glycated hemoglobin level of 8.1%. The patients were assigned to receive intensive therapy (targeting glycated hemoglobin level <6.0%) or conventional treatment (targeting between 7.0% and 7.9%). The study demonstrated that the use of intensive therapy to target normal glycated hemoglobin levels for 3.5 years increased mortality and did not significantly reduce major cardiovascular events [63].

The global findings of an attractive meta-analysis [64] showed modest gains of intensive glucose-lowering therapy on all-cause mortality and CV deaths. A 9% decrease or a 19% improvement in all-cause mortality and a 14% decrease or a 43% increase in cardiovascular deaths could not be ruled out. The benefit/risk profile of intensive hypoglycemic treatment in the prevention of macrovascular and microvascular events remains uncertain. The damage associated with severe hypoglycemia may outweigh the possible advantage of intensive blood glucose reduction treatment.

The less positive effects of intense glycemic control might be due to the side effects of antidiabetic treatment, including hypoglycemia events and weight gain. Hypoglycemia might be related to increased cardiovascular mortality, whereas weight gain is well known to be a risk factor of atherosclerosis. These two factors could have reduced the positive effect of lowering HbA1c in the UKPDS, PROactive, ADVANCE, and VADT studies and even contributed to the increased mortality in the ACCORD study. Another recently published study examining the relationship between poor glycemic control and polymorphism in the 9p21 locus (close to the CDKN2A-2B genes) highlighted an increased risk of developing coronary artery disease in carriers of two risk alleles [65]. Thus, by showing that the unfavorable effect of long-term hyperglycemia could be genetically related might help with personalizing diabetes control goals. The suggestion that the rapid correction of chronic hyperglycemia has a negative influence on macrovascular complications was supported by a recent descriptive study from 2022, which showed a clear increase in cardiovascular events following a dramatic reduction in HbA1c in hospitalized subjects with type 2 diabetes and high long-term glucose exposure [66].

The global outcome of this meta-analysis indicated a limited impact of intensive glucose-lowering treatment on all-cause mortality and cardiovascular deaths. A 9% decrease or a 19% increase in all-cause mortality and a 14% decrease or a 43% increase in cardiovascular deaths cannot be excluded. The benefit/risk ratio of intensive hypoglycemic therapy in the prevention of macrovascular and microvascular events remains unclear. The harm associated with severe hypoglycemia may outweigh the potential benefit of intensive blood glucose lowering therapy. More double-blind randomized controlled trials are needed to determine the most appropriate treatment approach for people with type 2 diabetes.

In Table 1, we summarize the results of the studies showing the possible impact of the intensive management of hyperglycemia on macrovascular complications in patients with diabetes.

Concerning the microvascular complications of diabetes, the deterioration of retinopathy in the case of intensive treatment for hyperglycemia is well known, as DR seems to be highly sensitive to intensive glucose therapy. This early worsening of DR has been reported in both type 1 [39,46] and type 2 diabetes [15,16,17,18,19,20,21,22,23,24,25,26,27,28,29,30,31,32,33,34,35,36,37,38,39,40,41,42,43,44,45,46,47,48,49,50,51,52,53,54,55,56,57,58,59,60,61,62,63,64,65,66,67] and mostly consists of more cotton wool spots, intraretinal microvascular abnormalities, and/or diabetic macular oedema, which indicates retinal ischemia. The proposed mechanisms behind this phenomenon include a decrease in the availability of cellular energy substrates, a reduction in the autoregulation of retinal circulation, and an increase in growth factors [40]. One prominent hypothesis used to explain this phenomenon is the upregulation of insulin-like growth factor 1 (IGF-1), which is a potent angiogenic and mitogenic hormone [68]. One important RCT showed that most people with type 1 diabetes have low levels of circulating IGF-1 [69]. This is, in part, due to the lack of portal venous insulin in these people, as IGF-1 secretion from the liver changes with the level of insulin. Intensive glycemic control leads to an increase in the amount of circulating IGF-1 with a brief spike, which may be linked to the effects of insulin on IGF-binding proteins and IGF-1 bioavailability [70]. An elevation in IGF-1 increases microvascular permeability and the effects of angiogenic factors [71]. IGF-1 has been shown to induce the existence of a vascular endothelial growth factor, a central angiogenic factor in the pathogenesis of DR, and to reinforce its downstream effects [72]. In animal models, the intravitreal injection of IGF-1 results in microvascular abnormalities resembling those of DR, including basement membrane thickening, vasodilation, vascular tortuosity, microaneurysms, intraretinal hemorrhages, and neo-vascularization [73,74,75,76].

Diabetic neuropathy seems to be the most sensitive microvascular complication associated with intensive hyperglycemia treatment. The pathophysiological mechanism behind this treatment-related pathology known as TIDN is not yet fully understood. Its symptomatology is short-lived, although the intensity of symptoms is directly related to the magnitude of the reduction in the HbA1c levels. A reduction of more than 5% over a 6-month period induces a relative risk of TIDN of more than 35% with the presentation of disseminated symptoms over the patient’s entire body [37,38]. The deterioration of nephropathy following intensive treatment for hyperglycemia seems to be marginal and is described in a very limited way in the cohorts of patients who developed TIDN. In our review, we shed light on a novel association between the intense correction of hyperglycemia and the onset of neuroarthropathy following the significant reduction in the HbA1c levels observed in two cohorts [57,58]. The evolution of the HbA1c levels prior to the discovery of CN seems to point to the cause of the inflammation, which is considered to be the spark triggering CN [53].

## 5. Conclusions

Our objective in this literature review was to focus on the link between intensive hyperglycemia treatment and the onset or worsening of certain macrovascular and microvascular complications in people living with diabetes, while acknowledging the necessity of hyperglycemia correction. At present and into the future, the management of diabetes will be marked by the democratization of effective therapeutic agents, such as closed-loop and hybrid subcutaneous insulin delivery systems, which will have a significant effect on the correction of the HbA1c levels [61,77]. Indeed, the benefit of these systems is their use of personalized algorithms where the progressive control of hyperglycemia can be chosen as an alternative to its rapid and non-gradual correction.

## Figures and Tables

**Table 1:** Results of the UKPDS, PROactive, ADVANCE, and ACCORD studies, looking at the impact of intensive hyperglycemia management on macrovascular complications in patients with diabetes.

Impact of the Intensive Management of Hyperglycemia	Reference	Stroke	Myocardial Infarction	Cardiovascular Mortality	Arteriopathy of Lower Limbs
UKPDS	[11]	No Impact	Reduction	No Impact	Not evaluated
PRO active	[12]	No Impact	No Impact	No Impact	Not evaluated
ADVANCE	[13]	Reduction	No Impact	No Impact	Not evaluated
ACCORD	[10]	No Impact	Reduction	Reduction	Reduction

## Data Availability

Not applicable.
